# Characteristics of Patients with Occupational Asthma in Türkiye, a Developing Country

**DOI:** 10.5334/aogh.5251

**Published:** 2026-05-18

**Authors:** Merve Demirci Atik, Nur Acar, Aylin Güngör Çifci, Arif Hikmet Çİmrİn

**Affiliations:** 1 Department of Occupational Disease, Faculty of Medicine, Dokuz Eylul University, İzmir, Turkey

**Keywords:** asthma, exposure, metal, occupational asthma, Türkiye

## Abstract

*Background:* Occupational asthma (OA) accounts for 10–25% of all adult-onset asthma cases. Prolonged exposure without avoidance worsens the prognosis of asthma. Diagnosis plays a key role in treatment by identifying risk factors and preventing exposure. Previous studies have shown that the most common causative substances may vary depending on the characteristics of each country, and these substances may result in different clinical presentations. This study aimed at evaluating the clinical features and characteristics of workplace exposures among OA patients diagnosed in an Occupational Disease Clinic in Türkiye.

*Methods:* In this cross-sectional study, data were collected retrospectively from clinical records by three experts using a standardised data registration form. Descriptive statistics for OA cases were presented. The chi-square test and the Mann–Whitney *U* test were used for comparative analysis.

*Results:* The mean time from symptom onset to OA diagnosis for 104 OA cases was nearly four years (46.75 months, ±56.20). High-molecular-weight (HMW) agents were responsible in 22.1% of cases. Skin prick test positivity and a history of atopy were significantly higher in individuals exposed to HMW agents (*p*-values: 0.008 and <0.001, respectively). One in five (20.2%) OA patients had exposure to metal dust, fumes or welding fumes, and two-thirds (67.3%) were employed in the industrial manufacturing sector.

*Conclusion:* OA is underdiagnosed, particularly in developing countries, and recognition of occupational causality is often delayed. Metal exposure, as indicated by the results, can be a significant contributor to OA in Türkiye. Identifying exposure potentials in line with local economic characteristics may raise awareness.

## Introduction

The prevalence of asthma has been defined as 1–29% in different countries, and it is estimated that as many as 300 million people suffer from asthma worldwide [1]. It is known that 10–25% of adult-onset asthma cases are occupational asthma (OA) [2, 3]. Although the reported annual incidence of OA is relatively higher in industrialised countries (18 per 100,000 population compared to 2 per 100,000 in developing countries), it is often underdiagnosed, poorly managed and inadequately compensated all around the world [4]. In patients of OA, prolonged exposure is often unavoidable, primarily due to low awareness, diagnostic challenges and individual financial barriers [5, 6]. Therefore, frequency of asthma attacks, drug use, and hospital admissions are higher in these patients [7]. It has been shown that the health costs of OA cases are 10 times higher than those of non-work-related asthma (non-WRA) patients [5]. Briefly, it is important to recognise OA cases and to prevent workplace exposures. Thus, it will improve individuals’ quality of life, decrease disease-related morbidity and mortality and reduce economic burdens such as health expenditures, workforce losses and layoffs [2, 3, 8].

In recent years, the phenotypic approach has become important in the concept of OA [9, 10]. OA can be classified into two main groups: allergic OA and non-allergic OA, also known as irritant-induced OA [3]. Additionally, it is emphasised that there may be differences in underlying pathophysiologies and clinical presentation depending on the molecular weight of the sensitising agent and the immune response [9]. On the other hand, the high-risk occupations and causative substances may vary depending on each country’s economic activities [4]. In most industrialised countries, the main causes of asthma include isocyanates, cereal flour/grain dust, welding fumes, and wood dust [9]. In contrast, in developing countries, although the causative agents are less consistent due to inadequate surveillance systems, cleaning products and pesticides are reported as prominent substances [4]. As well as in Türkiye, a developing country, comprehensive surveillance data remain lacking despite the publication of various case series [11–13]. However, it is well established that the proportion of the industrial manufacturing sector in Türkiye is higher than that observed in European countries [14], and the metal industry is at the forefront of the main production areas [15].

This study aimed at evaluating the potential exposures and the clinical characteristics of OA cases diagnosed in an occupational disease clinic in Türkiye, a developing country, as it is crucial to predict exposure based on local economic characteristics for both clinical considerations and prevention [4, 16].

## Materials and Methods

**Figure 1:**
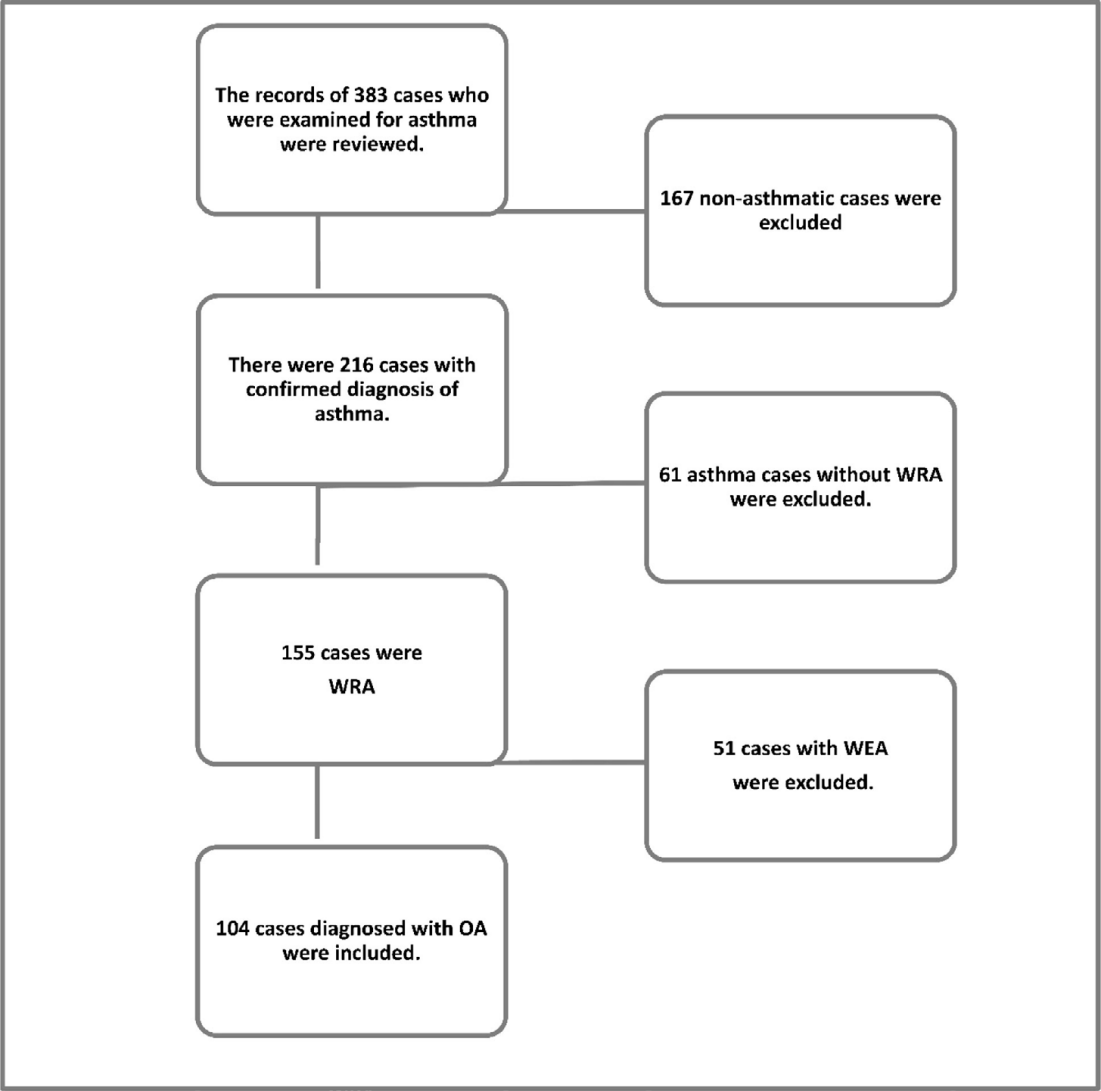
Diagram of selection of the study population. WRA: Work-Related Asthma. WEA: Work-Exacerbated Asthma. OA: Occupational Asthma.

This research was designed as a cross-sectional study. The ethical approval (2020/18-13) was obtained from the local committee, and Institutional permission was provided. The clinical records of 383 patients referred to Dokuz Eylul University Hospital’s Occupational Disease Clinic with a pre-diagnosis of asthma between 2013 and 2019 were reviewed retrospectively. There were 216 cases with a confirmed asthma diagnosis. Occupational association could not be demonstrated in 61 of them, and they were grouped as non-WRA. Out of 155 patients with work-related asthma (WRA), 51 had work-exacerbated asthma (WEA) and 104 had OA diagnosis. One hundred four cases diagnosed with OA were included in the study (Figure 1).

The variables of the study were demographic features of the patients, such as gender, age, smoking habits, job sectors and the substances they were exposed to, and clinical characteristics, such as symptoms, findings of physical examination, history of atopy, latency period (time from exposure to symptoms) and objective tests supporting the diagnosis. The data were collected by three occupational health experts using a standard registration form developed by the researchers. Exposures were determined based on statements from individuals and literature about the relevant sector. The responsible substances were categorised by molecular weight as high-molecular-weight (HMW) agents (>10 kDa) and low-molecular-weight (LMW) agents (<10 kDa), in accordance with the literature [9, 17]. Sectoral classification was described as three primary sectors: agriculture, industry and services, which typically refer to the global economic structure [18].

### Definitions and diagnostic criteria

**Diagnostic criteria for asthma:** Asthma is a heterogeneous disease usually characterised by chronic airway inflammation. It is defined by a history of variable, recurrent respiratory symptoms, such as wheeze, shortness of breath, cough and chest tightness, with variable expiratory airflow limitation [1]. In this study, the Global Initiative for Asthma criteria were used to confirm variable airflow limitation in a patient who has respiratory symptoms [1]. Variable airflow limitation was confirmed through the reversibility test, bronchial provocation test (BPT) or by demonstrating the diurnal variability of peak expiratory flow (PEF). The pulmonary function tests were performed using a Jaeger Master Screen spirometer device by a single technician, adhering to the related guidelines [19].

- **Reversibility test:** The criterion of >200 mL and >12% increase in FEV1 was used in the spirometric measurement performed 10–15 minutes after inhalation of 400 mcg salbutamol compared to the pre-bronchodilator measurement [1].
- **Nonspecific BPT:** To demonstrate nonspecific bronchial responsiveness, the methacholine challenge test was performed using the five-breath dosimeter protocol due to the ATS protocol [20].
- **Diurnal PEF variability:** A variability of >10% was considered diagnostic in measurements performed twice a day with the same Peakflowmeter for one week. ‘Day’s highest minus day’s lowest’ is divided by the average [1].

**Diagnostic criteria for OA:** ACCP (American College of Chest Physicians) criteria were used [3, 8]. It was confirmed that, in case all items A, B, C and D described below are present, this applies.

- Diagnosis of asthma
  And
- Onset of symptoms after entering the workplace
  And
- Association between symptoms of asthma and work
  And
- One or more of the following criteria to demonstrate a relationship between bronchial hyperreactivity and work environment:
  1. Identification of exposure to an agent known to cause OA in the workplace.
  2. Significant difference in PEF monitoring (PEF should be recorded at least four measurements per day; during two weeks of work and two weeks of rest) [3].
  3. Significant difference between working and resting nonspecific BPT (work-rest BPT) (more than 3 times difference in metacholine provocative dose) [3].
  4. Positive specific BPT to an agent to which the patient was exposed in the work environment.
  5. History of reactive airway dysfunction syndrome (RADS), characterised by the onset of symptoms after irritant exposure.

In this diagnostic algorithm, only ‘A+B+C+D1’ shows poor precision for diagnosing OA, while the others are more precise.

**RADS:** It is characterised by asthma-like symptoms occurring within the first 24 hours after inhalation of an acute high-level irritant in a patient with no prior asthma symptoms. The definition of RADS was first developed by Brooks et al. It is still used today with the concept of ‘acute onset irritant-induced asthma’ [21, 22].

**WEA:** It is defined as pre-existing or coincidental asthma that is exacerbated by a workplace-related stimulus [5]. As the diagnostic criteria, of the ACCP criteria, only the A (confirmation of the asthma diagnosis) and C (relationship between asthma symptoms and work done) items must be present together [3, 8].

**WRA:** The concepts of OA and WEA together are defined as WRA [5, 23].

**Non-WRA:** It is defined as asthma that does not develop or worsen due to exposures in the work environment [24].

**History of atopy:** It was defined as having any familial or personal history of an allergic disease, such as allergic rhinitis, conjunctivitis, urticaria, asthma or anaphylaxis.

**Skin prick test (SPT):** It was performed on patients with a history of atopy who consented. SPT was conducted using common aeroallergens on the volar side of the lower arm. The procedure was standardised using histamine as a positive control and glycerinated saline as a negative control. SPT results were considered positive if the wheal diameter was 3 mm or greater after subtracting the diameter of the negative control wheal, following the relevant protocols [25].

## Statistical Analysis

Descriptive statistics in the study were presented as numbers, percentages, means (±SD) and medians (min–max) values. Normality of the data was assessed using the Shapiro–Wilk and Kolmogorov–Smirnov tests. In comparative analysis, the chi-square test was used to compare proportions, and the Mann–Whitney *U* test was used to compare the difference between two means. Significance was accepted as *P* < 0.05. Analyses were performed with SPSS version 24 (IBM Corp., Armonk, NY).

## Results

The mean age of 104 cases with OA was 37.4 (±6.4) years, with a median of 37 (ranging from 21 to 52). Sixty-five and a half per cent of the cases involved males. The mean length of employment for the cases was 177.1 (±88.7) months, with a median of 168 months (8–450). The mean duration from symptom onset to OA diagnosis was 46.75 (± 56.20) months, with a median of 24 months (range 0–372). Median latency period, excluding four cases of RADS, was 60.0 (0–348) months, while the mean was 81.09 (±77.9).

The most common symptom of the cases was dyspnoea (93.3%). The others were cough (54.8%), wheezing (10%) and chest tightness (10%). The diagnosis of OA was confirmed in 17 cases (16.3%), based solely on relevant medical history, as functional assessments were not optimally diagnostic. Consequently, the diagnostic certainty in these 17 cases is lower than that in the remaining cohort. Diagnostic details of all cases are presented in Table 1.

**Table 1:** Characteristics of patients diagnosed with occupational asthma.

	NUMBERS (*N*) (*N* = 104)	PERCENTAGES (%) (100%)
**Gender**		
Male	68	65.4
Female	36	34.6
**Symptoms**		
Shortness of breath	97	93.3
Cough	57	54.8
Wheezing	12	11.5
Chest tightness	10	9.6
**Physical examination**		
Normal auscultation findings	47	45.2
Prolonged expiration	14	13.5
Rhonchus	43	41.3
**Smoking status**		
Never smoked	39	37.5
Ex-smoker	27	26.0
Current smoker	38	36.5
**History of atopy**		
Yes	31	29.8
No	73	70.2
**Concomitant chronic diseases**	49	47.1
Lumbar disc herniation	8	7.7
Mental health problems	7	6.7
Hypertension	4	3.8
Allergic rhinitis	4	3.8
Gastroesophageal reflux	4	3.8
**Diagnostic criteria of occupational asthma^a^**		
Relevant history^b^	17	16.4
Relevant history^b^ + work/rest_BPT	14	13.5
Relevant history^b^ + PEF monitoring	44	42.3
Relevant history^b^ + PEF monitoring + work/rest_BPT	25	24.0
Relevant history^b^ + RADS history	4	3.8

Abbreviations: BPT, bronchial provocation test; PEF, peak expiratory flow; RADS, reactive airway dysfunction syndrome.

^a^ In all cases, the diagnosis of asthma was confirmed first.

^b^ Relevant history: It must be a history that includes all three of the following criteria.
Onset of symptoms after entering the workplace. Association between symptoms of asthma and work. Identification of exposure to an agent known to cause occupational asthma in the workplace.

RADS was present in four cases. The first two cases were cleaning staff, the third was a chemical technician at a polyester factory and the fourth was a pickle manufacturing worker. One of the cleaning staff described an occupational accident due to exposure to pool chemical (sodium dichloroisocyanurate) and the other to sodium hypochlorite. A chemistry technician had a work accident with styrene monomer, and the pickle manufacturing worker experienced RADS symptoms after exposure to intense citric acid and sodium hydroxide. In all cases, symptoms had started on the same day, hours after exposure. Asthma was confirmed by nonspecific BPT in two cases and by diurnal PEF variability in the other two cases. The irritant and corrosive effects of all these agents on the respiratory system have been described in the literature [26]. The accidents they reported had occurred, respectively: one and four years before the hospital admission for the cleaning workers, six years prior to the chemical technician’s admission and eight years prior to the pickle manufacturing worker’s admission.

**Figure 2:**
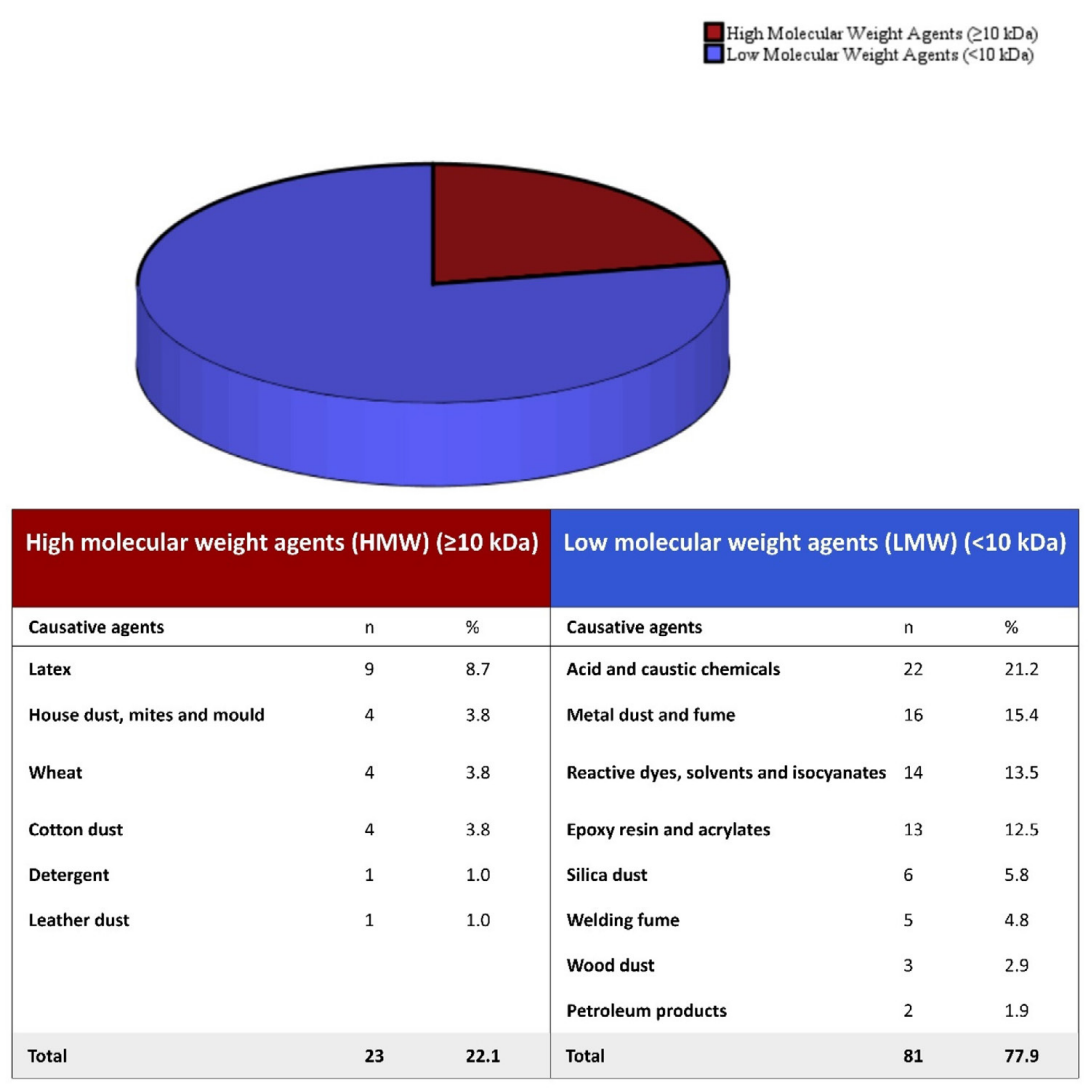
Distribution of occupational asthma cases by exposure agents.

Two-thirds of individuals were working in the industry sector. Most of them were in metalworking processes (19.2% of cases, including 12.5% in metal casting or cutting work and 6.7% in welding work). No individual was working in agriculture. The majority of those employed in the service sector have worked in healthcare (10.6%) and cleaning (9.6%) (Table 2). According to the causative exposures, HMW were found to be responsible in 22.1% of the cases. LMW, which may have both irritant and sensitising properties, were found to be responsible for 77.9% of the cases, mostly in the industry sector (79% of LMW). Acidic and caustic chemicals accounted for 21.2% of OA cases. Among all cases, 15.4% had exposure to metal dust or fumes, and 4.8% were exposed to welding fumes in their workplaces, amounting to a total of 20.2%. Therefore, it can be concluded that metals and welding fumes were one of the main exposures responsible for causing OA among our patients (Figure 2).

**Table 2:** Distribution of patients diagnosed with occupational asthma by sectors.

	NUMBERS (*N*) (*N* = 104)	PERCENTAGE (%)
**Service sector**	34	32.7
Health services	11	10.6
Cleaning services	10	9.6
Restaurant–bakery workers	4	3.8
Dental technicians	3	2.9
Laboratory workers	3	2.9
Others	3	2.9
**Industry sector**	70	67.3
Metal casting–cutting works	13	12.5
Textile manufacturing	8	7.7
Welding	7	6.7
Furniture manufacturing	7	6.7
Plastic and packaging manufacturing	7	6.7
Automotive manufacture and repair	5	4.8
Ceramic manufacturing	5	4.8
Chemical manufacturing	4	3.8
Food manufacturing	4	3.8
Wind energy panel manufacturing	4	3.8
Leather manufacturing	3	2.9
Artificial stone manufacturing	3	2.9
**Agriculture sector**	0	0

No significant difference was observed between the HMW and LMW exposure groups regarding age, latency period, smoking status and physical examination findings. Female sex, a history of atopy, SPT positivity and employment in the service sector were significantly more prevalent among individuals exposed to HMW allergens (Table 3).

**Table 3:** Comparison of patient characteristics based on the molecular weight of asthma-inducing agents.

	HMW	LMW	*P*-VALUE
**Age *years, means (±SD)***	39.13 (6.2)	36.88 (6.4)	*0.190*
**Latency period *months, means (±SD)***	87.57 (61.1)	79.16 (82.6)^c^	*0.207*
**Gender, *n* (%, row)**			*<0.001*
Male	7 (10.3)	61 (89.7)	
Female	16 (44.4)	20 (55.6)	
**Symptoms, *n* (%, row)**			
Shortness of breath^b^	21 (21.6)	76 (78.4)	0.647^a^
Cough^b^	9 (15.8)	48 (84.2)	0.087
Wheezing^b^	4 (33.3)	8 (66.7)	0.458^a^
Chest tightness^b^	1 (10.0)	9 (90.0)	0.452^a^
**Physical examination, *n* (%, row)**			0.852
Normal findings	10 (21.3)	37 (78.7)	
Abnormal findings	13 (22.8)	44 (77.2)	
**Smoking Status, *n* (%, row)**			0.502
Never smoked	10 (25.6)	29 (74.4)	
Ever smoked (current or ex)	13 (20.0)	52 (80.0)	
**History of atopy, *n* (%, row)**			*<0.001*
Yes	16 (51.6)	15 (48.4)	
No	7 (9.6)	66 (90.4)	
**Presence of allergic rhinitis, *n* (%, row)**			0.033^a^
Yes	3 (75.0)	1 (25.0)	
No	20 (20.0)	80 (80.0)	
**Skin prick test, *n* (%, row)** ^d^			0.008
Positive	13 (65.0)	7 (35.0)	
Negative	3 (20.0)	12 (80.0)	
**Sectors, *n* (%, row)**			*<0.001*
Service	17 (50.0)	17 (50.0)	
Industry	6 (8.6)	64 (91.4)	

*Note:*

^a^ Fisher’s exact test was used because the cell count exceeded 20% with an expected value of less than 5.

^b^ In the chi-square test, only rows with symptomatic individuals are shown, omitting asymptomatic data for simplicity.

^c^ RADS cases (*n* = 4) were excluded from this analysis.

^d^ Only 35 of the cases underwent a skin prick test.

## Discussion

Workplace exposures associated with OA typically differ across countries based on local economic characteristics [4]. According to the results of this study, it was observed that one in five cases (20.2%) involved exposure to metal or welding fume, and two-thirds of the OA cases (67.3%) were associated with the industrial manufacturing sector. It is known that the metal industry is at the forefront of Türkiye’s industrial sector, ranking as the second largest producer of scrap metal processing and iron steel in Europe and eighth globally [15]. Another hospital-based study conducted in Türkiye was previously reported; the ratio of metalworkers and welders was high (16.9% and 12.5%, respectively) among cases with WRA [11]. The data from another reference centre in Istanbul, the country’s most populous city, have shown that the leading causative job sector among cases diagnosed with WRA was also metalworking [27]. Moreover, field studies in our country have also been frequently conducted in the metal sector. For instance, in a foundry factory, Kayhan et al. reported a 16.78% prevalence of OA [13]; meanwhile, in a bicycle factory, 22% of welders were found to have OA by Temel et al. [12]. In contrast, according to the literature, the forefront hazards differ in developed countries [4]. Based on surveillance data in the USA, metal and welding exposure was seen in only 53 (1.6%) of 2209 asthma cases, while the most common agents were chemicals (*n* = 523, 16.1%), mineral and organic dusts (*n* = 477, 14.7%) and cleaning products (*n* = 440, 13.5%) [10]. Data from Germany and France indicate that the most common workplace asthmatic agents are flour, isocyanates and latex. Furthermore, the highest-risk groups in these countries are mainly found in the service sector, including bakery workers and hairdressers [28, 29]. Similarly, a multicentre study in Europe reported that among 1180 cases diagnosed with OA via specific BPT, the most common occupational exposures were flour cereals (*n* = 369, 31.3%), isocyanates (*n* = 206, 17.4%) and persulphate salts (*n* = 78, 6%) [9].

To date, more than 400 causative agents of OA have been documented in the literature [30, 31]. These causative agents related to OA are categorised into two main groups based on molecular weight: HMW agents, LMW agents, along with other irritants [3, 9, 17, 30, 31]. Our results revealed that the majority (77.9%) of OA cases in our clinic had been exposed to LMW agents and irritants in the workplace, including acidic or caustic substances, solvents, reactive chemicals, metals and welding fumes. The pathogenesis of asthma caused by LMW agents remains largely unclear. LMW agents mainly sensitise through cellular immune–mediated pathways or by acting as haptens, specifically inducing immunoglobulin E (IgE) [30, 32]. Some of the LMW chemicals can also cause respiratory irritation [3, 30]. As a result of irritation, the potential role of non-immunological mechanisms, such as epithelial injury, airway wall remodelling, oxidative stress or neurogenic inflammation, is under debate [6, 32]. In a large retrospective cohort study by Vandenplas et al. [9], conducted in 2019, the characteristics of asthma patients were compared according to the molecular weight of sensitised agents. They observed differences in asthma symptoms between subjects exposed to LMW agents and those exposed to HMW agents. Those exposed to LMW agents were more likely to experience chest tightness and daily sputum production at work. In contrast, individuals exposed to HMW agents were more likely to suffer from work-related rhinitis, conjunctivitis and urticaria. Additionally, they found a slightly shorter latency period in subjects with OA caused by LMW agents compared to those exposed to HMW agents. In our study, the latency period of patients exposed to LMW was also shorter than that of others. However, our results were not statistically significant, likely due to small sample sizes similar to those in previous studies [6, 33]. In fact, it is more widely believed that the length of the latent period of asthma indeed depends on the nature of the substance, not just its molecular weight [10]. More importantly, a longer latency period without exposure avoidance indicates a worse prognosis for persistent and severe asthma [10, 17]. Regarding HMW agents, these substances may elicit IgE-mediated type I hypersensitivity reactions [9, 11, 28, 31]. According to our study results, SPT positivity and a history of atopy were significantly higher among individuals exposed to HMW. Similarly, numerous publications have demonstrated the association between atopy, defined as having at least one positive SPT response to common aeroallergens, and sensitisation to HMW agents [6, 9, 17]. It is also known that HMW sensitisers, including plant and animal proteins, are generally encountered among certain service-sector workers, such as bakers, laboratory animal workers and healthcare workers [3, 32]. According to our study results, most of those exposed to HMW agents (73.9%) worked in the service sector, as expected (*p* < 0.001).

It is known that OA cases are underdiagnosed, especially in developing countries [9, 37]. Individuals with persistent exposure likely have numerous physician visits or emergency department visits, and recognition of occupational causality is often delayed. A study by Breton and colleagues showed that patients with WRA were 4.8 times more likely to visit the emergency department at least once and 2.5 times more likely to visit their physician for an asthma exacerbation in the previous 12 months than subjects with non-WRA [7]. In our study, the median time from symptom onset to OA diagnosis was 24 months (range 0–372), with an average of nearly four years (46.75 months, ±56.20). It is consistent with other research [5, 34]. According to Poonai et al., the main reasons for the delay in OA diagnosis were lack of enquiry about work-relatedness by the primary care physician (41%), fear of losing work time (37%) and fear of forced job loss (33%) [34]. Furthermore, the reported delays in secondary care were closely linked to the challenges encountered in completing necessary investigations [34]. When it comes to investigations in secondary care, the specific BPT, as a gold-standard test for sensitiser-induced OA [3, 35], remains significantly underutilised, even in European countries [35]. Although being a reference centre, a specific BPT could not be performed in any of our cases. Because of the complex and specialised technical requirements, its use is mainly restricted to a handful of academic centres. Moreover, access to nonspecific BPT, which has a high negative predictive value of 97.7% for OA when conducted at least once in the workplace [36], is limited in our country and available only in a few laboratories nationwide. Furthermore, other supplementary tests for diagnosing sensitiser-induced OA, such as induced sputum eosinophilia, exhaled nitric oxide (FeNO) or specific IgE (skin tests or serum specific IgE) [9], are also not widely accessible in daily practice in most health centres, creating barriers to confirming OA diagnosis. Our study results indicate that PEF monitoring during periods at and off work settings was the most commonly used method, utilised in two-thirds of cases, to support the occupational causality of asthma in patients. This method, achieving 64% sensitivity and 77% specificity, is relatively cost-effective and widely used in practice, enabling its application without laboratory facilities [3]. Additionally, in 17 cases (16.3%) at our clinic, the causality between occupation and asthma was determined based solely on relevant medical history (‘A+B+C+D1’ criteria of ACCP) [3] due to issues such as patient compliance/preferences or technical limitations that prevented further diagnostic testing. Previous studies have also highlighted the predictive value of nearly 75% of a comprehensive medical history in diagnosing OA, which may often be sufficient, particularly in cases caused by irritants [3, 37]. Given that almost half of our patients (45.2%) had normal auscultation findings during clinical presentation, it is clear how important a detailed medical history and symptom enquiry are for diagnosing asthma because there may be no physical examination clues at all [1]. The physician’s awareness is crucial in primary care [34].

The treatment process has also been poorly managed for the reasons mentioned above. Particularly in developing countries, if measures such as elimination, substitution or avoidance are not possible at the workplace, many employees are compelled to change their working sectors [5]. Indeed, most of them quit their jobs before even being diagnosed with asthma, because of their work-related symptoms. Due to this condition, also known as the healthy worker effect, it is often challenging to identify the true number of OA patients [38]. It is expected that the detected cases were those who continued working under the same conditions, likely due to financial difficulties, despite experiencing disturbing symptoms. For our country, another main problem of managing OA is the insufficient registration system. The recording of occupational diseases is maintained solely by the social insurance institution in Türkiye when compensation is involved. For this reason, the recorded cases are even below the numbers that can actually be diagnosed. For example, only 165 cases of OA were recorded nationwide in Türkiye over 10 years from 2013 to 2023 [39]. However, it is very clear that the burden of workplace exposures, which is known to be responsible for 16% of asthma cases, cannot be limited to just a few instances [2].

## Strengths and Limitations of the Study

It should be emphasised that, as one of the few reference occupational disease clinics in the country, our centre receives many patient admissions. Therefore, it can be assumed that the data, particularly from the country’s Aegean region, are well reflected in our study. However, as a limitation, the results cannot be generalised to the country as a whole due to the single-centre design. Another limitation of the study is that data were collected retrospectively from the records. Related agents were categorised based on the most probable exposure estimate, considering the patients’ statements and the literature. Therefore, it was not possible to clearly evaluate the simultaneous exposures and the definite phenotypes (such as sensitiser- or irritant-induced) from the records. Additionally, the diagnosis of OA in some patients, based on their medical history, posed another limitation of the study. However, this ambiguity was mitigated by clearly outlining the diagnostic criteria of ACCP (‘A+B+C+D1’ criteria). Lastly, it should be emphasised that our study also lacks data regarding the agricultural sector. This is because, in our country, agricultural work is carried out individually by villagers rather than by corporate firms. The exposures in their work and living environments are intertwined. Therefore, they visit general clinics more frequently rather than occupational disease clinics.

## Conclusion

Especially in developing countries, cases of OA remain underdiagnosed, and defining the occupational causality is often delayed. It is crucial to raise awareness among both workers and healthcare providers. Studies reflecting local economic activities in the countries may be helpful for accurate and simple exposure estimates.
